# Analysis of correlation between dynamic contrast-enhanced ultrasound and angiogenesis activity of renal cell carcinoma

**DOI:** 10.1055/a-2513-1054

**Published:** 2025-09-15

**Authors:** Xiuyun Lu, Jianqing Ye, Xiuwu Pan, Shaojun Chen, Liang Zhang, Ying Wang, Juan Cheng, Jiaying Cao, Li Wei, Xingang Cui, Yi Dong

**Affiliations:** 1 91603 Ultrasound, Xinhua Hospital Affiliated to Shanghai Jiaotong University School of Medicine, Shanghai, China; 2 91603 Urology, Xinhua Hospital Affiliated to Shanghai Jiaotong University School of Medicine, Shanghai, China

**Keywords:** tumor, urinary, surgery, ultrasound

## Abstract

**Purpose:**

To investigate the potential correlation of dynamic contrast-enhanced ultrasound (DCE-US) with angiogenesis activity of renal cell carcinoma (RCC).

**Materials and Methods:**

Patients with surgery resection and histopathologically proven RCC lesions were included. B-mode ultrasound (BMUS) and contrast-enhanced ultrasound (CEUS) were performed one week before surgery. SonoVue was injected as the contrast agent. VueBox (Bracco, Italy) was used for the quantitative analysis. According to the histopathological and immunohistochemical results, patients were classified into two groups: active angiogenesis and inactive angiogenesis. Time intensity curves (TICs) and quantitative parameters were compared between two groups.

**Results:**

From July 2023 to November 2023, a total of 50 patients (13 females and 37 males, mean age 61.1±11.1 years) were included. The mean size of the lesions was 39.4±2.7 mm. Patients were classified into the active angiogenesis group (n=30) and the inactive angiogenesis group (n=20). On BMUS, 68.0% (34/50) of RCCs were visualized as hypoechoic lesions with ill-defined borders and irregular shapes (P>0.05). During cortical phase of CEUS, 72.6% (23/30) of RCCs with active angiogenesis were visualized with hyperenhancement (P=0.027). Only 30.0% (9/30) of RCCs with active angiogenesis showed hypo-enhancement in the parenchymal phase (P>0.05). Compared to the inactive angiogenesis group, TICs of the active angiogenesis group revealed faster and greater enhancement in the cortical phase, slower decline during the parenchymal phase, and an increased area under the curve. Among quantitative parameters, the active angiogenesis group showed the higher ratio of wash-in rate and wash-in perfusion index (P<0.05).

**Conclusion:**

DCE-US analysis has potential value in predicting angiogenesis activity in RCC lesions.

## Introduction

Renal cell carcinoma (RCC) is one of the most common malignant renal tumors and is projected to be one of the top 10 causes of cancer-related deaths by 2040 1 2 . In RCC patients, the angiogenesis activity of the tumor in histopathology specimens is evaluated to determine the vascular perfusion in the tumor 3 . Angiogenesis activity is defined microscopically by pivotal molecular targets including vascular endothelial growth factor (VEGF) and microvascular density, which was assessed by CD31 or CD34 4 . The angiogenesis activity of the tumor has potential value as a predictive biomarker for assessing the prognosis of RCC patients and formulating precise therapy plans 5 . The 5-year survival rate for RCCs with inactive angiogenesis was 92.5%, while that of RCCs with active angiogenesis was low and was only 12% in patients with metastasis 6 . Therefore, whether the tumor has active angiogenesis or not affects the further therapeutic schemes and the prognosis.

According to the latest European Federation of Societies for Ultrasound in Medicine and Biology (EFSUMB) guidelines and recommendations, contrast agents are totally vascular agents following intravenous injection, and they can highlight the macro- and micro-vascular systems on contrast-enhanced ultrasound (CEUS) 7 . Due to the strengths such as real-time scan, safety, and convenience, CEUS has been widely used for evaluating microvascular perfusion in RCC lesions 8 . For the majority of RCC characterization, it is vitally important to evaluate cortical hyperenhancement and washout in the parenchyma phase 9 .

Dynamic contrast-enhanced ultrasound (DCE-US) enables the quantitative analysis of microvascular perfusion based on time-intensity curves (TICs) and quantitative parameters 10 . By quantitative analysis of perfusion and angiogenesis of solid renal masses, DCE-US has been reported to have the potential for differential diagnosing between benign and malignant lesions 11 . As a dynamic, safe, and repeatable imaging method, DCE-US has been used to quantify the microvascular perfusion of RCC lesions and to monitor therapeutic response induced by anti-angiogenic therapy 12 . It is recognized as a potential biomarker of response and as a tool to optimize therapy in individual patients 13 . However, little has been published on the possibility of DCE-US in predicting angiogenesis activity before surgical resection.

The purpose of our study was to investigate the potential value of DCE-US in evaluating the angiogenesis activity of RCC lesions.

## Patients and methods

### Patient group

This retrospective study was approved by the institutional review board. Informed consent was waived. The procedure was carried out in accordance with the Helsinki Declaration.

**Fig. 1:**
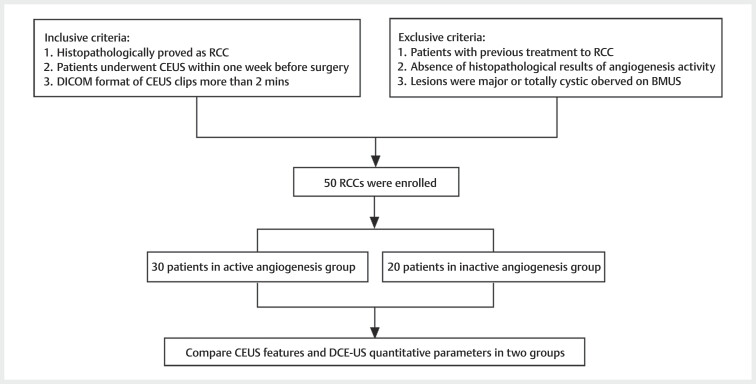
Flowchart of study (RCC: renal cell carcinoma; BMUS: B-mode ultrasound; CEUS: contrast-enhanced ultrasound; DCE-US: dynamic contrast-enhanced ultrasound).

The inclusion criteria were: 1) Patients were diagnosed with RCCs based on histopathological results of surgery resection; 2) Patients underwent CEUS examination one week before surgery; 3) DICOM format of CEUS clips more than 2 mins were available; and 4) solid RCC lesions could be clearly found on BMUS Fig. 1 .

The exclusion criteria were: 1) Patients with previous treatment, such as chemotherapy, radiotherapy, etc.; 2) Patients without definite histopathological results of angiogenesis activity (CD31, CD34); 3) The lesions observed on BMUS were majorly or totally cystic.

### Contrast-enhanced ultrasound (CEUS) examination

All RCC patients underwent conventional ultrasound and CEUS examinations equipped with the 5C-1 convex array transducer (ACUSON Sequoia; Siemens Medical Solutions, USA). The location, size, internal homogeneity, and blood flow were observed on conventional ultrasound. A bolus of 1 ml of contrast agent (SonoVue, Bracco, Italy) was injected via the cubital vein and followed by a 5 ml saline flush. According to the EFSUMB guidelines, the enhancement degrees and enhancement patterns of RCCs during the cortical phase and parenchyma phases were recorded 7 . Two-minute cine CEUS loops were stored in DICOM format for further analysis.

### Dynamic contrast-enhanced ultrasound (DCE-US) quantitative analysis

The CEUS clips were analyzed using the VueBox software (Bracco, Italy). According to the EFSUMB guidelines and recommendations on the use of DCE-US, two regions of interest (ROIs) were positioned in the RCC lesions and the surrounding renal cortex at the same depth 14 . TICs were created and compared between the two groups. After curve fitting, various DCE-US quantitative parameters were acquired, including peak enhancement (PE), wash-in area under the curve (WiAUC), rise time (RT), mean transit time (mTT), time to peak (TTP), wash-in rate (WiR), wash-in perfusion index (WiPI=WiAUC/rise time), washout area under the curve (WoAUC), wash-in and washout area under the curve (WiWoAUC), fall time (FT), and washout rate (WoR) Table 1 . The ratios of these quantitative parameters between RCC lesions and the surrounding renal cortex were calculated. Quantitative parameters were compared between the active angiogenesis group and the inactive angiogenesis group.

**Table:** **Table 1** Dynamic contrast-enhanced ultrasound (DCE-US) quantitative parameters.

Abbreviation	Specific meanings
**WiAUC**	wash-in area under the curve
**WoAUC**	washout area under the curve
**WiWoAUC**	wash-in and washout area under the curve
**PE**	peak enhancement
**RT**	rise time, the time from injection to the beginning of enhancement
**TTP**	time to peak, the period between arrival of contrast agent in the ROI to PE
**mTT**	mean transit time, the period between 50% and PE
**FT**	fall time
**WiPI**	wash-in perfusion index
**WiR**	wash-in rate
**WoR**	washout rate

### Histopathological analysis

The unstained histology slides were stained with hematoxylin and eosin (H&E). Angiogenesis activity was evaluated by anti-CD31 and anti-CD34 antibodies, which were used as targets to visualize endothelial cells in immunohistochemistry. According to the final histopathological results, RCC patients were separated into 2 groups: the active angiogenesis group and the inactive angiogenesis group. The active angiogenesis group was defined as CD31 (+) and CD34 (+), CD31 (+) and CD34 (-), CD31 (-) and CD34 (+). The inactive angiogenesis group was CD31 (-) and CD34 (-).

### Statistical analysis

Continuous variables were compared using student’s *t* -test or Mann-Whitney U-test. Pearson’s *χ* ^2^ test or Fisher test was used for categorical variables. The statistical analyses were conducted utilizing the SPSS software (version 25.0) and GraphPad Prism 7 (GraphPad Software, Inc.). *P* -value<0.05 was considered statistically.

## Results

### Patient characteristics

From July 2023 to November 2023, 50 patients with histopathologically confirmed RCC lesions were included in this study consisting of 13 males and 37 females (mean age: 61.1±1.6 years) Table 2 . According to the final histopathological results, 30 patients (60.0%) were assigned to the active angiogenesis group, while 20 patients (40.0%) were in the inactive angiogenesis group. Table 1 displays a comprehensive description of patient demographics and pathological distribution in two groups.

**Table:** **Table 2** Patient demographics and biochemical distribution of renal cell carcinoma.

Variables	Active angiogenesis group (n=30)	Inactive angiogenesis group (n=20)	*P* -value
**Sex**			0.035
**Female**	11 (36.7%)	2 (10.0%)	
**Male**	19 (63.3%)	18 (90.0%)	
**Age (year) †**	62.9±10.7	60.3±11.8	0.472
**WHO/ISUP grade**			0.858
**G1+G2**	20 (66.7%)	13 (65.0%)	
**G3+G4**	7 (23.3%)	4 (20.0%)	
**Biochemical indicators**			
**RBC (/LP) ‡**	5.0 (2.0, 15.0)	4 (2.5, 5.5)	0.112
**Ca (mmol/L) ‡**	2.2 (2.1, 2.3)	2.23 (2.2, 2.3)	0.260
**BUN (mmol/L) ‡**	6.5 (5.4, 8.9)	6.2 (6.0, 8.0)	0.051
**Cr (μmol/L) ‡**	61.0 (47.0, 91.0)	65.0 (50.0, 81.0)	0.491
**UA (μmol/L) ‡**	281.0 (252.0, 348.0)	375.0 (262.0, 490.3)	0.985
**BUN:CREA ‡**	0.10 (0.09, 0.13)	0.11 (0.08, 0.13)	0.303
**GFR (ml/min) ‡**	102.6 (73.5, 118.6)	107.9 (87.2, 118.5)	0.567
**LDH (U/L) ‡**	181.0 (171.0, 193.0)	165.5 (147.8, 203.5)	0.162
**Pathology diagnosis**			0.143
	ccRCC (90.0%)	ccRCC (70.0%)	
	pRCC (3.3%)	pRCC (20.0%)	
	FH-deficient RCC (3.3%)	EVT (5.0%)	
	ELOC-mutated RCC (3.3%)	cRCC (5.0%)	

RBC: red blood cell of urine under the microscope; Ca: calcium; BUN: blood urea nitrogen; Cr: creatinine; UV: uric acid; BUN: blood urea nitrogen; CREA: creatinine; GFR: glomerular filtration rate; LDH: lactate dehydrogenase; ccRCC: clear cell renal cell carcinoma; pRCC: papillary renal cell carcinoma; EVT: eosinophilic vacuolated tumor; ELOC-muted RCC: Elongin C-mutated renal cell carcinoma; cRCC: chromophobe renal cell carcinoma.

### Comparison of conventional ultrasound and contrast-enhanced ultrasound (CEUS) features

**Fig. 2:**
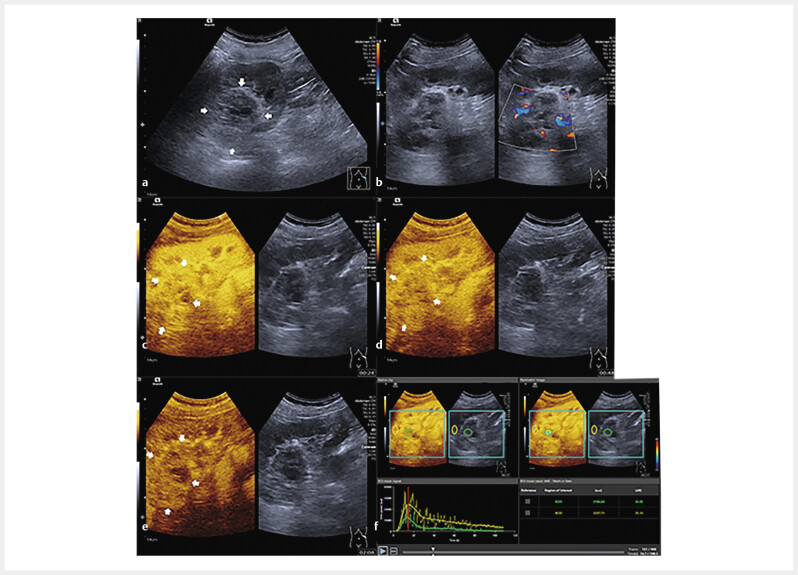
A case of renal cell carcinoma (RCC) with inactive angiogenesis. A male patient aged 70 years with accidentally detected solid renal tumor. On B-mode ultrasound, a hypoechoic lesion with a size of 54 mm and an unclear margin was detected in the upper pole of the left kidney ( **a** ). No blood flow signal was found on the color Doppler flow image ( **b** ). After injecting contrast agents, the lesion showed heterogeneous iso-enhancement in the cortical phase of contrast-enhanced ultrasound ( **c** ), and hypo-enhancement in the parenchymal phases ( **d, e** ). Two regions of interest (ROIs) were set in the lesion (green one) and the surrounding renal parenchyma (yellow one). Compared with the time-intensity curve (TIC) of the renal parenchyma, the TIC with green color showed that the lesion reached peak intensity slower, with lower peak enhancement and a decreased area under the curve ( **f** ). The lesion was confirmed by surgery resection and histopathological results as a clean cell renal cell carcinoma with CD31 (-), CD 34 (-).

On conventional BMUS, 23 RCC lesions were in the left kidney, while 27 lesions were in the right kidney. The mean sizes of RCC lesions were 39.4±3.7 mm and 39.4±3.9 mm in the active and inactive angiogenesis groups, respectively. On BMUS, most of the RCC lesions (34/50, 68.0%) were visualized as hypoechoic lesions with ill-defined borders and irregular shapes ( *P* >0.05) Table 3 , Fig. 2 3 .

**Fig. 3:**
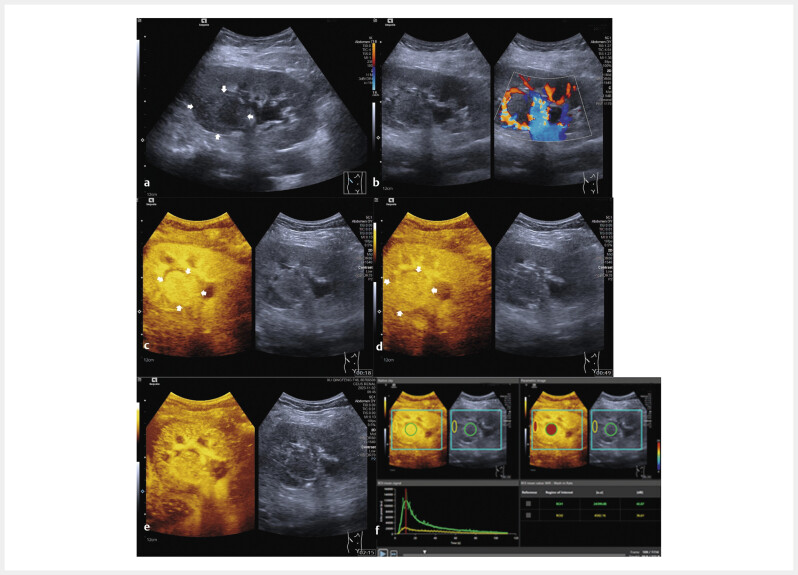
A case of renal cell carcinoma (RCC) with active angiogenesis. A male patient aged 46 years with accidentally detected solid renal tumor. On B-mode ultrasound, a hypoechoic lesion with a size of 34 mm and an unclear margin was detected in the middle upper part of the right kidney ( **a** ). Multiple linear blood flow signals were found on the color Doppler flow image ( **b** ). After the injection of contrast agents, the lesion showed heterogeneous hyperenhancement in the cortical phase ( **c** ) and the parenchymal phase ( **d, e** ). Two regions of interest (ROIs) were set in the lesion (green one) and the surrounding renal parenchyma (yellow one). Compared with the time-intensity curve (TIC) of the renal parenchyma, the TIC with green color showed that the lesion reached peak intensity faster, with higher peak enhancement and an increased area under the curve ( **f** ). The lesion was confirmed by surgery resection and histopathological results as a clean cell renal cell carcinoma with CD31 (+), CD 34 (+).

**Table:** **Table 3** Comparison of conventional ultrasound features between renal cell carcinoma lesions with active and inactive angiogenesis.

Imaging features	Active angiogenesis group (n=30)	Inactive angiogenesis group (n=20)	*P* -value
**B-mode ultrasound**			
**Laterality**			0.774
**Left**	13 (43.3%)	10 (50.0%)	
**Right**	17 (56.7%)	10 (50.0%)	
**Lesion size (mm)**	39.4±3.7	39.4±3.9	0.929
**Echogenicity**			1.000
**Hypoechoic**	20 (66.6%)	14 (70.0%)	
**Isoechoic**	3 (10.0%)	1 (5.0%)	
**Hyperechoic**	7 (23.3%)	5 (25.0%)	
**Border**			0.687
**Clear**	5 (16.7%)	2 (10.0%)	
**Ill-defined**	25 (83.3%)	18 (90.0%)	
**Shape**			
**Regular**	6 (20.0%)	1 (5.0%)	0.219
**Irregular**	24 (80.0%)	19 (95.0%)	
**Color Doppler flow imaging**			
**Color flow signals**	20 (66.7%)	14 (70.0%)	1.000
**Resistance index**	0.65±0.03	0.60±0.03	0.139

During the cortical phase of CEUS, most RCC lesions with active angiogenesis (72.6%, 23/30) showed hyperenhancement, while most RCC lesions with inactive angiogenesis (60.0%, 12/20) showed hypo- or isoenhancement ( *P* =0.027) Table 4 , Fig. 2 3 . Subsequently, 30.0% (9/30) of RCCs with active angiogenesis and 45.0% (9/20) of RCCs with inactive angiogenesis showed hypoenhancement in the parenchymal phase ( *P* >0.05). The ROC curve was plotted for enhancement pattern during the cortical phase in differentiating angiogenesis activity. The sensitivity, specificity, area under the curve (AUC), and 95% confidence interval (CI) of the enhancement pattern during the cortical phase was 76.7%, 60.0%, 0.673 (0.516, 0.829).

**Table:** **Table 4** Comparison of contrast-enhanced ultrasound features between renal cell carcinoma lesions with active and inactive angiogenesis.

Imaging features	Active angiogenesis group (n=30)	Inactive angiogenesis group (n=20)	*P* -value
**Cortical phase**			0.027
**Hypoenhancement**	4 (13.3%)	5 (25.0%)	
**Isoenhancement**	3 (10.0%)	7 (35.0%)	
**Hyperenhancement**	23 (76.7%)	8 (40.0%)	
**Parenchymal phase**			0.614
**Hypoenhancement**	9 (30.0%)	9 (45.0%)	
**Isoenhancement**	14 (46.7%)	7 (35.0%)	
**Hyperenhancement**	7 (23.3%)	4 (20.0%)	

### Comparison of dynamic contrast-enhanced ultrasound (DCE-US) time-intensity curves (TICs)

When comparing the TICs of the two groups, the active angiogenesis group had faster and greater enhancement in the cortical phase Fig. 2 3 . Moreover, the active angiogenesis group took longer to wash out in the parenchymal phase than the inactive ones. An increased area under the curve (AUC) was found in the active angiogenesis group compared to the inactive group Fig. 2 3 .

### Comparison of dynamic contrast-enhanced ultrasound (DCE-US) quantitative parameters

**Fig. 4:**
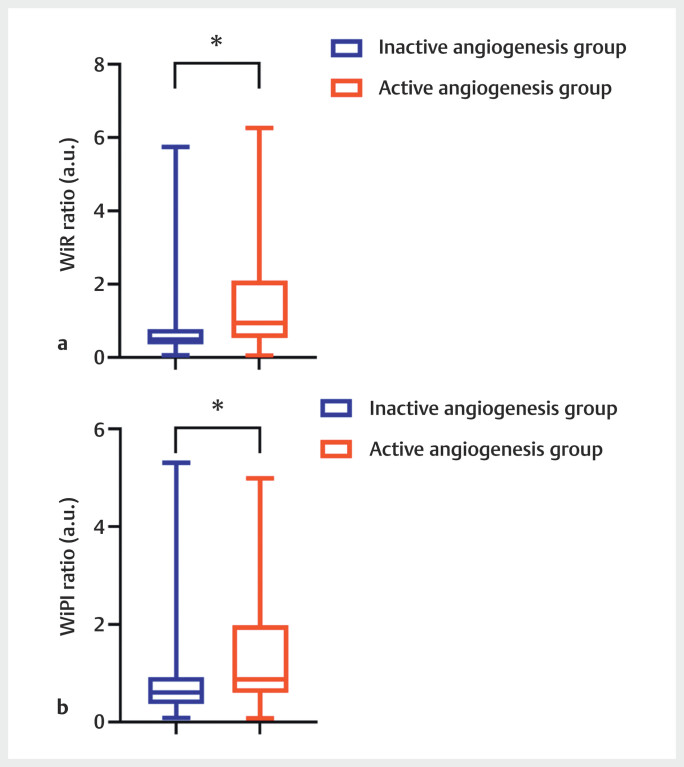
Comparison of dynamic contrast-enhanced ultrasound quantitative parameters between the active and the inactive angiogenesis groups (WiR: wash-in rate; WiPI: wash-in perfusion index; *: P<0.05).

**Fig. 5:**
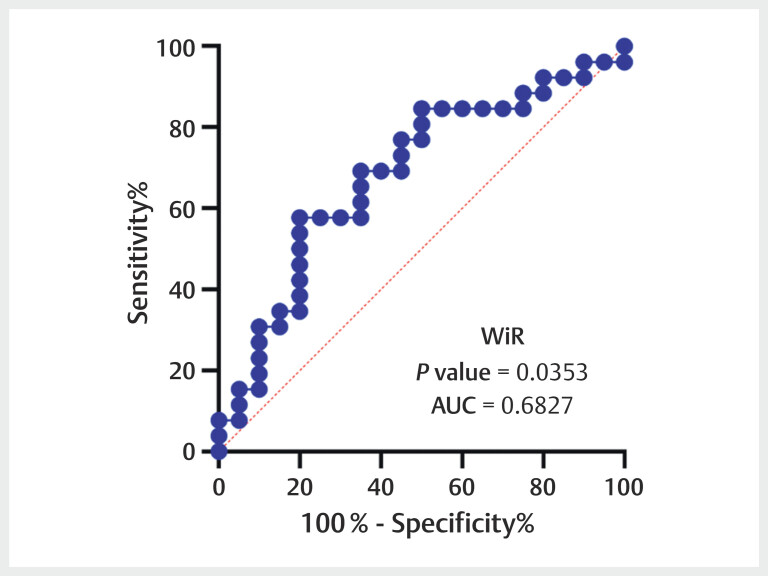
The receiver operating characteristic (ROC) curve of the wash-in rate (WiR) ratio in predicting the angiogenesis activity of renal cell carcinoma. The area under the ROC curve of it yielded a value of 0.683 (95% CI: 0.524–0.841).

DCE-US quantitative parameters were created and compared after curve fitting. Among all DCE-US parameters, the ratio of WiR and WiPI were higher in the active angiogenesis group than in the inactive angiogenesis group ( *P* <0.05) Table 5 , Fig. 4 . The ROC curve was plotted for these two parameters in differentiating angiogenesis activity Fig. 5 6 . The sensitivity, specificity, AUC, and 95% CI of the ratio of WiR were 57.7%, 80.0%, 0.683, and 0.524–0.841, respectively. In addition, the sensitivity, specificity, AUC, and 95% CI of the ratio of WiPI were 73.1%, 65.0%, 0.671 and 0.510–0.832, respectively. Combining the DCE-US analysis and CEUS features improved the diagnostic efficiency significantly. The sensitivity, specificity, AUC, and 95% CI of combined analysis were 88.5%, 60.0%, 0.737, and 0.528–0.891, respectively. The combination of DCE-US and CEUS showed better diagnostic efficiency than CEUS alone: sensitivity (88.5% vs. 76.7%), specificity (60.0% vs. 60.0%), area under the curve, and 95% confidence interval (0.737 [0.528–0.891] vs. 0.673 [0.516–0.829]). Taking 0.485 as the cut-off value, DCE-US was able to provide added value in predicting the angiogenesis activity of RCCs. We added these accordingly in the results section of our revised manuscript.

**Fig. 6:**
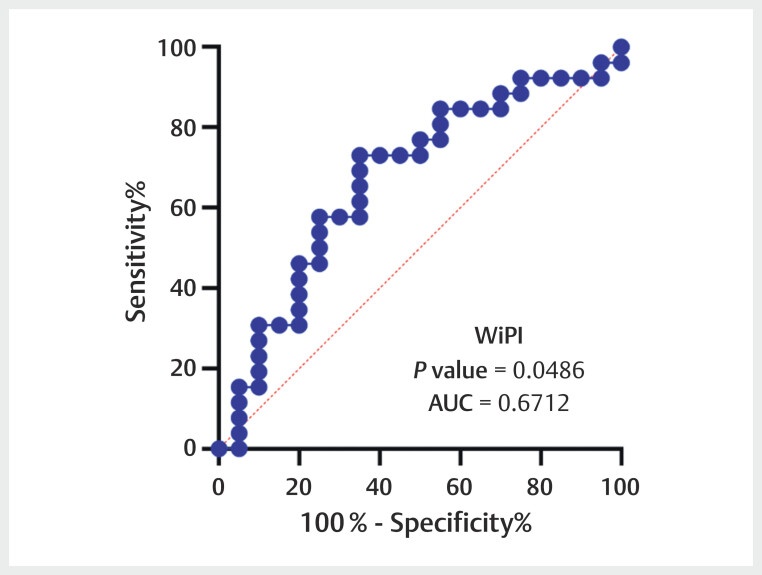
The receiver operating characteristic (ROC) curve of the wash-in perfusion index (WiPI) ratio in predicting the angiogenesis activity of renal cell carcinoma. The area under the ROC curve of it yielded a value of 0.671 (95% CI: 0.510–0.832).

**Table:** **Table 5** Quantitative dynamic contrast-enhanced ultrasound parameters based on histopathologic classification.

Variables	Active angiogenesis group (n=30)	Inactive angiogenesis group (n=20)	*P* -value
**PE (a.u)**	25853.5 (13784.7, 47874.0)	14606.5 (4570.2, 39567.4)	0.076
**WiAUC (a.u)**	148101.8 (65391.8, 284669.3)	89445.4 (28043.0, 164429.8)	0.144
**RT (s)**	6.7 (5.5, 11.9)	7.7 (6.2, 11.3)	0.240
**mTT (s)**	43.4 (34.2, 56.1)	44.7 (32.7, 54.9)	0.877
**TTP(s)**	11.6 (8.6, 17.5)	11.9 (10.7, 17.2)	0.478
**WiR (a.u)**	5543.8 (3158.3, 9762.7)	2512.9 (581.0, 8425.5)	0.054
**WiPI (a.u)**	18068.8 (8865.5, 30052.1)	9217.1 (2892.0, 24915.5)	0.080
**WoAUC (a.u)**	314493.5 (126436.9, 540243.8)	177349.8 (63557.5, 365937.4)	0.073
**WiWoAUC (a.u)**	487445.7 (187794.2, 820510.0)	266795.2 (96885.0, 523819.1)	0.080
**FT (s)**	16.1 (8.7, 26.6)	14.9 (11.2, 24.5)	0.650
**WoR (a.u)**	1859.1 (972.2, 4985.2)	872.3 (171.0, 3789.3)	0.121
**PE ratio (a.u)**	0.8 (0.6, 2.0)	0.6 (0.4, 0.9)	0.054
**WiAUC ratio (a.u)**	1.0 (0.6, 1.9)	0.6 (0.4, 1.8)	0.156
**RT ratio (s)**	1.0 (0.8, 1.3)	1.1 (1.0, 1.2)	0.215
**mTT ratio (s)**	0.9 (0.6, 1.3)	0.9 (0.7, 1.4)	0.807
**TTP ratio (s)**	1.0 (0.9, 1.2)	1.1 (1.0, 1.2)	0.215
**WiR ratio (a.u)**	0.9 (0.5, 2.1)	0.5 (0.4, 0.8)	0.035
**WiPI ratio (a.u)**	0.9 (0.6, 2.0)	0.6 (0.4, 0.9)	0.049
**WoAUC ratio (a.u)**	1.1 (0.6, 1.9)	0.6 (0.4, 1.9)	0.258
**WiWoAUC ratio (a.u)**	1.1 (0.6, 1.9)	0.6 (0.4, 1.9)	0.231
**FT ratio (s)**	1.0 (0.7, 1.6)	1.2 (1.0, 1.4)	0.223
**WoR ratio (a.u)**	1.0 (0.4, 2.1)	0.4 (0.2, 0.9)	0.076

Data are median (25%, 75% quartiles). Qualitative variables are analyzed using Mann-Whitney U-test. PE: peak enhancement; WiAUC: wash-in area under the curve; RT: rise time; mTT: mean transit time; TTP: time to peak; WiR: wash-in rate; WiPI: wash-in perfusion index; WoAUC: washout area under the curve; WiWoAUC: wash-in and washout area under the curve; FT: fall time; WoR: washout rate.

## Discussion

Imaging modalities may shed some light on the quantification of microvascular blood flow in RCC lesions, which is related to the angiogenesis activity of the lesion and relative clinical treatment strategies 15 16 . Previously reported studies revealed the preoperative value of computed tomography (CT) and magnetic resonance imaging (MRI) in predicting microvascular density 17 18 19 . Although CT and MRI have high spatial resolution, their contrast agents have extravascular distribution, which limits the evaluation of microvascular perfusion 20 . According to the EFSUMB guidelines, CEUS provide important information for evaluating microvascular perfusion in tumors 7 . Measured using the immunohistochemical technique, angiogenesis activity is crucial for tumor growth and metastasis and is a factor in determining the surgical method 18 21 . To clinically optimize the treatment plan and improve the prognosis of patients, the angiogenesis activity of the tumor should be systemically evaluated.

The angiogenesis activity, assessed by CD31 and CD34 immunohistochemistry, has been largely studied and its importance in progression is widely accepted 22 23 . In our study, the differences in CEUS features between RCC patients with active and inactive angiogenesis were analyzed. During the cortical phase of CEUS, 76.7% (23/30) of RCCs with active angiogenesis and 40.0% (8/20) of RCCs with inactive angiogenesis exhibited hyperenhancement. Subsequently, more RCCs in the inactive angiogenesis group exhibited hypoenhancement in the parenchymal phase than in the active angiogenesis group (45.0% of the inactive angiogenesis group vs. 30.0% of the active angiogenesis group). A previous study reported that high peak intensity in the cortical phase of CEUS had the potential to predict microvascular density of renal pelvic urothelial carcinomas 24 . Renal pelvic urothelial carcinomas with higher microvascular density exhibited higher peak enhancement intensity on CEUS. However, only qualitive information regarding wash-in and washout in RCCs could be found on CEUS, which cannot show the correlation between CEUS with angiogenesis activity of RCCs precisely. Thus, quantitative analysis needs to be performed for determining its potential value in evaluating angiogenesis activity of RCC lesions.

Dynamic contrast-enhanced ultrasound is reliable for quantitatively displaying the phases of progressive increase and subsequent phases of slow decrease in the enhancement of lesions 25 26 . By creating TICs of real-time perfusion, DCE-US shows the dynamic wash-in and washout differences between benign and malignant renal tumors 27 . In our study, while comparing the difference in microvascular perfusion between the active angiogenesis group and the inactive angiogenesis group, a significant difference was found in the TICs of DCE-US. The TICs of RCCs in the active angiogenesis group showed higher peak intensity than the inactive ones, faster wash-in and increased AUC. It is consistent with the results of the previous study, which shows the role of DCE-US in the preoperative prediction of an RCC’s invasiveness 28 . Compared to noninvasive RCCs, TICs of invasive RCCs showed faster wash-in, higher peak intensity, and an increased AUC. Based on the hemodynamic evaluation of RCC lesions, TICs might provide additional value in predicting angiogenesis activity before surgery 21 .

Further quantitative parameters originated from TICs offer us quantitative assessments of microvascular perfusion in renal tumors 13 29 . The ratios of quantitative parameters between RCCs as well as the surrounding renal parenchyma were calculated to lessen the effect of varying patients and the depths of lesions. In our study, when comparing the active and the inactive angiogenesis groups, the DCE-US parameters, including WiR and WiPI, were significantly different. These two parameters were significantly higher in the active angiogenesis group, which could be explained by the invasive effect of the active angiogenesis group. Combining DCE-US analysis and CEUS features improved the diagnostic efficiency in angiogenesis activity significantly. Previous studies analyzed DCE-US quantitative parameters for predicting the invasion of RCCs, including PE, WiR, WiPI, and WoR 28 . Moreover, DCE-US perfusion parameters such as FT, RT TTP, mTT, and AUC were reported to be valuable from differentiating renal tumors as well as evaluating therapeutic efficacy in RCCs 12 29 30 . Intravenous injection of contrast agent can show the perfusion and regression of renal microvascular perfusion, which can be clearly evaluated by quantitative analysis software 14 31 . The results of WiR and WiPI on DCE-US seem to provide added value in predicting the angiogenesis activity of RCCs.

There were various limitations in our study. The sample size is relatively small in this retrospective study. Therefore, further data collection is required to evaluate the presence of angiogenesis activity of RCCs.

## Conclusion

DCE-US with quantitative analysis has the potential to predict angiogenesis activity in RCC lesions in future diagnosis. WiR and WiPI might be valuable quantitative parameters.
